# Lipid metabolism in multiple myeloma: pathogenesis, therapeutic opportunities, and future directions

**DOI:** 10.3389/fonc.2025.1531928

**Published:** 2025-03-05

**Authors:** Huiquan Wang, Jiafeng Zhang, Hefei Ren, Lei Chen, Jigang Ren, Chang Liu, Hongkun Wu, Lin Zhou

**Affiliations:** Department of Laboratory Medicine, Shanghai Changzheng Hospital, Naval Medical University, Shanghai, China

**Keywords:** multiple myeloma, lipid metabolism, therapeutic targets, tumor, hematology

## Abstract

Background: Multiple myeloma (MM) is a complex hematological malignancy characterized by the clonal expansion of plasma cells in the bone marrow. Emerging studies have emphasized the importance of lipid metabolism, which is closely associated with the survival, proliferation, and drug resistance of tumor cells. The hypoxic environment in the bone marrow (BM) contributes to metabolic reprogramming in MM cells, including alterations in metabolite levels, changes in metabolic enzyme activity, and metabolic shifts. Cancer cells possess the ability to adapt their metabolism in order to fulfill their continuously increasing energy demands. In this review, we will discuss the alterations in lipid metabolism during the development of MM, and their reciprocal interactions with the tumor microenvironment.

## 1 Introduction

In recent years, the role of lipid metabolism in MM has become a focal point of research. MM cells exhibit imbalances in lipid processing, which is manifested in multiple aspects (1–5). Firstly, there are alterations in lipogenesis. Key enzymes like fatty acid synthase (FASN) are over - expressed (6, 7), leading to increased *de novo* fatty acid synthesis. This provides the necessary lipids for rapid cell membrane expansion during MM cell proliferation. Secondly, lipid uptake and trafficking are disrupted. MM cells enhance their ability to take up fatty acids from the microenvironment, especially from bone marrow adipocytes (BMAs) (8). BMAs, as energy reservoirs, are exploited by MM cells. They secrete fatty acids that are avidly taken up by MM cells through specific transporters such as fatty acid - binding proteins (FABPs) and fatty acid transporter proteins (FATPs) (9, 10). Thirdly, the tricarboxylic acid (TCA) cycle, which is closely related to lipid metabolism, is also perturbed (11, 12). This leads to an imbalance in energy production and metabolite generation, further fueling the survival and growth of MM cells.Moreover, lipid metabolism in MM also has a profound impact on the tumor microenvironment (TME). Lipid - induced oxidative stress in the TME can damage the DNA, proteins, and lipids within MM cells, but at the same time, MM cells adapt to this stress by upregulating antioxidant defense mechanisms. In addition, lipid - rich tumor - associated macrophages (TAMs) play a crucial role in immune evasion. M2 - polarized TAMs secrete immunosuppressive factors like IL - 10 and TGF - β, express immune checkpoint molecules such as PD - L1, and phagocytose immune cells, all of which contribute to creating an immunosuppressive environment that benefits MM cell survival (13).

The insights into lipid metabolism in MM have opened up new avenues for developing novel therapies. Targeting lipid metabolism has emerged as a promising strategy (5). For example, inhibitors of lipogenesis, such as FASN inhibitors, can disrupt the abnormal lipid synthesis in MM cells. By blocking FASN, the production of palmitate is reduced, which in turn disrupts the lipid raft structure and pro - survival signaling pathways like AKT/mTOR (14–16). This leads to the induction of apoptosis in MM cells. Another approach is to target lipid uptake. Inhibiting the transporters involved in fatty acid uptake can starve MM cells of essential lipid nutrients. For instance, interfering with the function of FABPs or FATPs can prevent MM cells from efficiently taking up fatty acids from BMAs. Furthermore, modulating the TCA cycle can also be a viable strategy. By regulating the key enzymes in the TCA cycle, the energy metabolism of MM cells can be disrupted, making them more vulnerable to treatment. In addition, lipid - based biomarkers and metabolic imaging techniques, combined with artificial intelligence, hold great potential for personalized medicine. They can help in early diagnosis, accurate prognosis, and predicting treatment responses, enabling more tailored treatment strategies for individual MM patients.

Despite the significant progress, there are still aspects of lipid metabolism in MM that remain under - explored (17). The interaction between lipid metabolism and the immune microenvironment is a complex and not fully understood area (1, 18). Although we know that lipid - induced oxidative stress and lipid - rich TAMs affect the immune response, the detailed molecular mechanisms underlying how lipid metabolism precisely regulates immune cell functions in the context of MM are unclear. For example, how the lipid composition of MM cells and the TME affects the activation, differentiation, and effector functions of T cells, B cells, and NK cells requires further investigation.

Moreover, the high heterogeneity of MM poses challenges in understanding lipid metabolism. Different MM subtypes may have distinct lipid metabolic profiles, and how to precisely target these differences for effective treatment is yet to be fully elucidated. Also, the crosstalk between different lipid metabolic pathways and their combined effects on MM cell biology and the TME are still not well - characterized. Addressing these unstudied aspects will be crucial for the development of more effective and targeted therapies for MM.

## 2 Imbalanced lipid metabolism in multiple myeloma

Multiple myeloma (MM) is a complex hematological malignancy, and imbalances in lipid metabolism has emerged as a fundamental characteristic, underlying various metabolic abnormalities that are central to its pathophysiology. MM cells exhibit a distinct profile of abnormal lipid metabolism, with alterations spanning multiple aspects of lipid homeostasis. This includes a significant upregulation of *de novo* lipogenesis, enhanced lipid uptake, abnormal formation of lipid droplets, and disrupted lipid trafficking processes (19).

### 2.1 Transcriptional and post-transcriptional regulation of lipid metabolism in MM

Emerging evidence highlights the critical role of transcriptional and post-transcriptional regulators in mediating lipid metabolic imbalances in multiple myeloma (MM). Sterol regulatory element-binding proteins (SREBPs), particularly SREBP-1c, act as master transcriptional regulators of lipogenic enzymes such as acetyl-CoA carboxylase (ACC) and fatty acid synthase (FASN), which are frequently upregulated in MM cells to sustain aberrant lipid biosynthesis (20). Post-transcriptional modulation by non-coding RNAs further amplifies lipid metabolic reprogramming. For instance, miR-27a-3p directly suppresses the expression of stearoyl-CoA desaturase (SCD), a key enzyme in monounsaturated fatty acid synthesis, thereby altering membrane fluidity and drug sensitivity in MM cells (21). Targeting these regulators—via pharmacological SREBP inhibitors or RNA-based therapies—represents a promising strategy to disrupt lipid dependency in MM, potentially enhancing therapeutic efficacy.

### 2.2 Mitochondrial-lipid metabolism link abnormalities in MM

Mitochondrial dynamics and bioenergetic dysfunction are increasingly implicated in MM-associated lipid metabolic alterations. Recent studies reveal imbalances of mitochondrial fission/fusion machinery in MM cells, characterized by reduced expression of fusion regulators and elevated activity of dynamin-related protein 1 (Drp1), a driver of mitochondrial fragmentation (22). Excessive mitochondrial fission impairs fatty acid β-oxidation (FAO) by disrupting cristae structure and electron transport chain efficiency, forcing MM cells to rely on *de novo* lipogenesis for survival. Intriguingly, pharmacological inhibition of Drp1 not only restores mitochondrial homeostasis but also suppresses lipogenic gene expression, suggesting a mechanistic link between mitochondrial dynamics and lipid anabolism (23). These findings position mitochondrial regulators as dual modulators of bioenergetic and lipid metabolic pathways in MM.

### 2.3 Adiponectin-mediated adipokine signaling in MM

Adipokines play a pivotal role in the intricate relationship between lipid metabolism and the progression of MM, with adiponectin being particularly prominent. Adiponectin is an adipokine deeply involved in fatty acid metabolism. In MM, plasma cells downregulate the expression of adiponectin. Adiponectin possesses anti - tumor properties, and one of its primary functions is to inhibit the production of IL - 6 (24–26). Under normal physiological conditions, an appropriate level of adiponectin can effectively regulate the immune microenvironment and suppress the growth and proliferation of tumor cells.

However, in MM patients, the reduction in adiponectin levels disrupts this balance. The decrease in adiponectin levels leads to the upregulation of IL - 6. IL - 6, in turn, promotes the growth and survival of myeloma cells, thereby driving disease progression. A profound understanding of the signaling pathways of adiponectin and other adipokines offers the potential for therapeutic innovation. By developing drugs that can mimic the functions of adiponectin or therapies that can upregulate adiponectin expression, it may be possible to block the tumor - promoting effects mediated by IL - 6. For example, researchers could design a specific agonist that mimics the binding of adiponectin to its corresponding receptor, thereby activating the downstream anti - tumor signaling pathways. Alternatively, a drug that can regulate gene expression to promote the synthesis of adiponectin and restore its normal level in the body could be developed. This would inhibit the growth of MM cells and open up new avenues for the treatment of MM (27–30).

### 2.4 Serum lipid level manifestations

One of the most tangible manifestations of the dysregulated lipid metabolism in Multiple Myeloma (MM) is the alteration in serum lipid levels. To comprehensively understand these changes, a multitude of well - designed clinical investigations have been meticulously carried out.

During the active phase of MM, a consistent finding is that patients exhibit lower high - density lipoprotein cholesterol (HDL - C) levels. This phenomenon has been firmly supported by a substantial amount of research data. In 14 independent studies (31–44), the HDL - C levels in patients with active MM were measured to range from 26 to 52.4 mg/dl. In contrast, the HDL - C levels in the control group, consisting of healthy individuals, spanned from 38.6 to 58 mg/dl. Simultaneously, triglyceride levels in MM patients are significantly elevated at the time of diagnosis and remain so throughout the active disease stage. This abnormal elevation of triglyceride levels not only reflects the derangement of lipid metabolism in the diseased state but also poses a potential threat to the cardiovascular health of patients. High levels of triglycerides are well - established as a major risk factor for cardiovascular diseases. When comparing different International Staging System (ISS) stages of MM with healthy individuals, significant differences in low - density lipoprotein cholesterol (LDL - C), HDL - C, and total cholesterol levels are clearly observable. For instance, in a large - scale study involving 307 MM patients, the prognostic significance of several plasma markers was comprehensively evaluated. Serum cholesterol, LDL, HDL, apolipoprotein A1 (Apo A1), and apolipoprotein B1 (Apo B1) all demonstrated significant statistical significance in the analysis of variance (ANOVA) (P<0.001, P<0.001, P = 0.002, P<0.001, and P<0.001, respectively). Serum ApoA1, in particular, showed significant differences between stage I and stage II, as well as between stage II and stage III (P = 0.007 and P = 0.002, respectively). Moreover, Apo A1 levels were significantly associated with progression - free survival (PFS) and disease - specific survival (CSS) (Hazard Ratio HR: 0.484 and 0.344, 95% Confidence Interval CI: 0.255 - 0.922 and 0.152 - 0.779, P = 0.028 and P = 0.011) (44). These results suggest that Apo A1 levels can serve not only as an important reference indicator for MM staging but also as a crucial biomarker for assessing the survival prognosis of patients. In another in - depth study focusing on the correlation between lipid expression levels and multiple myeloma, a potential causal relationship was discovered between triglyceride (TG) levels and MM risk (Odds Ratio OR: 0.67, 95% Confidence Interval CI: 0.46 - 0.98, P = 0.038) (45).

In summary, MM patients demonstrate distinct patterns of serum lipid level changes across different disease stages, including the active phase, remission stage, and various ISS stages. HDL - C levels decrease during the active phase, while triglyceride levels increase, and both return to normal during remission. Comparing different ISS - staged patients with healthy individuals reveals significant differences in multiple lipid indicators. Additionally, certain lipid - related biomarkers such as Apo A1 are closely associated with patient prognosis, and there is a potential causal link between TG levels and MM risk. These cumulative research findings not only contribute to a deeper understanding of the pathophysiological mechanisms of MM but also offer valuable lipid - related biomarkers for the diagnosis, staging, and prognosis assessment of MM, providing a robust theoretical basis for the formulation of clinical treatment strategies.

### 2.5 Association between obesity, dysregulated lipid metabolism, and the risk and progression of multiple myeloma

Obesity is an established risk factor for the development and progression of MM. Obesity leads to an increase in the number and volume of bone marrow adipocytes (BMAs) (46–48)., which is directly associated with an elevated risk of MM. In patients with monoclonal gammopathy of undetermined significance (MGUS), a precursor to MM, lipid - reactive clonal immunoglobulins are often present, and obesity in these patients further exacerbates the risk of progression to MM. In animal models, obesity - induced lipid imbalances and diet - induced obesity can promote the development of a myeloma - like syndrome.

In MM, abnormal lipid metabolism confers several advantages to cancer cells. The overproduction of lipids, enhanced lipid uptake, and altered lipid droplet dynamics contribute to the enhanced survival, proliferation, and energy generation of cancer cells. Additionally, dysregulated lipid metabolism disrupts the redox balance within MM cells, leading to increased oxidative stress and further tumor progression. MM cells can also reprogram BMAs, which impacts bone lesions and the tumor microenvironment. Due to promoter methylation, reprogrammed adipocytes have lower levels of peroxisome proliferator - activated receptor γ (PPARγ), and this reduction is associated with an inability to reverse bone lesions after disease remissionγ) (49).

Based on these associations, for high - risk patient populations such as obese individuals or those with MGUS, targeting lipid metabolism pathways could be a viable and effective therapeutic strategy. By developing drugs that target key lipid metabolism enzymes or modulating adipokine signaling pathways, it is possible to intervene in the development and progression of MM. For instance, inhibiting key enzymes involved in lipid synthesis can reduce the lipid resources available to cancer cells, thereby inhibiting their growth. Modulating adipokine signaling pathways, such as those related to adiponectin, is expected to restore the balance of the immune microenvironment and suppress the growth of tumor cells. This not only provides a new direction for the treatment of MM but also offers hope for improving the treatment outcomes of MM patients. Through precise intervention in lipid metabolism, more effective control and treatment of MM may be achieved.

## 3 Lipid metabolism and tumor microenvironment in multiple myeloma

### 3.1 Role of bone marrow adipocytes in the TME

**Figure 1:**
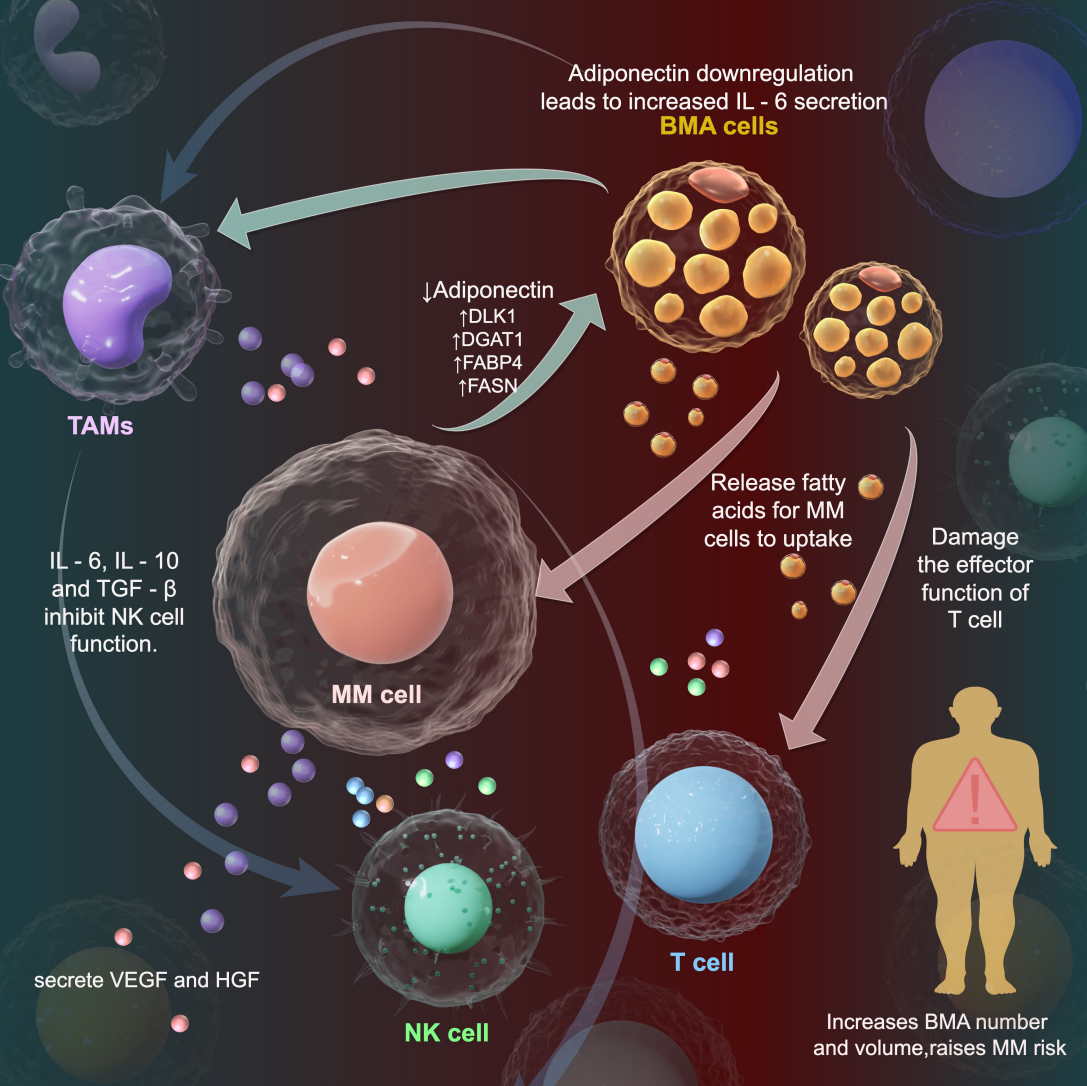
Interaction of myeloma cells, bone marrow adipocytes and tumor microenvironment in multiple myeloma. This diagram shows immune - regulatory and lipid - related mechanisms of multiple myeloma (MM) cells in the tumor microenvironment (TME). MM cells interact with bone marrow adipocytes (BMAs). BMAs release fatty acids for MM cells to make lipid droplets and extracellular vesicles. In MM patients, adiponectin (anti - tumor adipokine) level drops, while IL - 6 level rises, creating a microenvironment for myeloma cell growth. Oxidized fatty acids in vesicles enter tumor - infiltrating T cells, harming their functions.MM cells polarize tumor - associated macrophages (TAMs) into M2 type. M2 - TAMs secrete IL - 10 and TGF - β. IL - 10 suppresses the immune response, TGF - β weakens NK cell cytotoxicity, promoting tumor growth. Decreases, while the level of IL - 6 increases, facilitating the growth of myeloma cells.

As shown in **Figure 1**, the reprogramming of bone marrow adipocytes (BMAs) by multiple myeloma (MM) cells to manipulate the tumor microenvironment (TME) is a complex and crucial process. MM cells interact with BMAs, altering their gene expression and functional status (48). Bone marrow adipocytes (BMA) are cells in the bone microenvironment that have been implicated in promoting anti-apoptosis and multiple myeloma disease progression in MM cells by activating NF-κB signaling through IL-6 secretion (50). In one study, BMAs were found to have higher expression of adipocyte differentiation-related genes DLK1, DGAT1, FABP4, and FASN, which may lead to higher secretion of adipose-related factors by BMAs and promote MM cell proliferation and growth (6). An increase in FABP4 gene expression facilitates the binding and transportation of fatty acids within BMAs (6). In tumors with a high frequency of bone metastases, BMAs exert their pro-tumorigenic effects mainly through the regulation of genes related to lipid metabolism, including FABP4, PPARγ and CD36 (6, 51). BMAs have been found to secrete a number of molecules important for supporting MM cells in the bone marrow, and to directly recruit MM cells through monocyte chemotactic protein 1 and stromal cell-derived factor 1α (46). Once these fatty acids are taken up by MM cells, they can generate a large amount of adenosine triphosphate (ATP) through the β - oxidation pathway, providing energy support for the rapid proliferation of MM cells (52–54). Vascular endothelial growth factor (VEGF) and hepatocyte growth factor (HGF) are multifunctional cytokines that stimulate angiogenesis during tumor neovascularization. VEGF is secreted by MM cells, which induce MM cell proliferation and stimulate IL-6 expression by microvascular endothelial cells and bone marrow stromal cells (55, 56).

### 3.2 The crucial impact of lipid-induced oxidative stress in multiple myeloma

In the intricate pathological mechanism of multiple myeloma, lipid - induced oxidative stress plays a pivotal role, especially in promoting tumor cell growth and inhibiting immune cell functions. This process profoundly influences the development of the disease oxidative stress in MM cells (57).

Once the lipid metabolism in the tumor microenvironment (TME) becomes abnormal, a series of cascading reactions are triggered, with the generation of a large amount of reactive oxygen species (ROS) being a crucial starting point. When the ROS level rises sharply, an oxidative stress state ensues, creating a special environment for the growth of myeloma cells. In this environment, multiple signaling pathways within myeloma cells are abnormally activated. For example, the mitogen - activated protein kinase (MAPK) signaling pathway is activated by oxidative stress, which promotes the expression of genes related to myeloma cell proliferation, such as Cyclin D1. The over - expression of this protein accelerates the cell cycle process, enabling myeloma cells to divide and proliferate more rapidly, thus driving tumor growth.

The inhibitory effect of oxidative stress on immune cells is also remarkable. For T cells, lipid - induced oxidative stress disrupts their normal signal transduction process. The T - cell receptor (TCR) signaling pathway is inhibited under oxidative stress, making it difficult for T cells to be effectively activated. As a result, T cells cannot fully exert their cytotoxic effects and fail to recognize and kill myeloma cells in a timely manner. Natural killer (NK) cells are also severely affected. Oxidative stress reduces the expression of activation receptors on the surface of NK cells, such as NKp46, while increasing the expression of inhibitory receptors. This significantly impairs the cytotoxic function of NK cells, rendering them unable to effectively perform immune surveillance and elimination of myeloma cells.

Moreover, oxidative stress also affects the chemotaxis and migration abilities of immune cells. Under normal circumstances, immune cells can accurately migrate to the tumor site to exert immune functions under the guidance of chemokines. However, in the lipid - induced oxidative stress environment, the expression of chemokine receptors on the surface of immune cells is down - regulated, weakening their responsiveness to chemokines and preventing them from migrating normally into the tumor microenvironment. This further weakens the ability of the immune system to attack myeloma cells.

In multiple myeloma, lipid - induced oxidative stress plays a key role in promoting the development of the disease by promoting tumor cell growth and inhibiting immune cell functions. In - depth exploration of this mechanism is of great significance for developing more effective treatment strategies to break the growth advantage of tumor cells and restore the normal functions of immune cells.

### 3.3 Immune regulation by multiple myeloma cells through lipid pathways

In the complex pathophysiological landscape of myeloma development, the tumor microenvironment serves as a critical arena where multiple myeloma (MM) cells orchestrate a series of intricate processes to manipulate the immune system, with lipid - associated mechanisms playing a central role. Tumor-associated macrophages (TAM) are an important component of the bone marrow (BM) in multiple myeloma (MM) patients, accounting for approximately 10% of the BM (58). Apart from the well - studied interactions between MM cells and bone marrow adipocytes (BMAs), M2 - polarized tumor - associated macrophages (TAMs) emerge as key players in this immune - regulatory drama (59, 60). As is well known, the M1 TAM subtype can stimulate the Th - 1 immune response and exert an anti - tumor effect (13), while M2 TAM has low antigen - presenting ability and can promote tumor progression by inducing immunosuppression and angiogenesis (58, 61–65). However, MM cells can induce the polarization of TAM into the tumor - promoting M2 phenotype, causing them to secrete anti - inflammatory molecules, including IL - 10 and tumor growth factor (TGF) - β.

Tumor - infiltrating T cells have their effector functions impaired due to lipid peroxidation and mitochondrial stress triggered by lipid droplets and extracellular vesicles rich in oxidized fatty acids derived from MM cells (66). In this process, MM cells utilize the fatty acids taken up from BMAs to synthesize lipid droplets and extracellular vesicles. After the oxidized fatty acids carried by these substances enter tumor - infiltrating T cells, they disrupt the normal lipid metabolism balance within T cells, trigger mitochondrial stress responses, and ultimately lead to the inability of T cells to exert normal immune - killing functions. In multiple myeloma (MM), transforming growth factor-β (TGF-β) produced by plasma cells (67), regulatory T cells (Tregs) (68), or possibly myeloid - derived suppressor cells (MDSCs) (69) can downregulate natural killer (NK) cell activation receptors and impair NK cell function (70, 71). Elevated levels of interleukin - 6 (IL - 6) and interleukin - 10 (IL - 10) have also been observed in MM (72, 73). These cytokines act as growth factors for plasma cells and promote the development of an NK - resistant tumor phenotype by inhibiting NK cell activity (74, 75). In essence, by regulating the lipid metabolism of TAMs and the subsequent release of immunosuppressive factors, MM cells skillfully manipulate the immune response within the tumor microenvironment. There are complex lipid - metabolism - related connections between MM cells and various cells in the microenvironment (13, 76, 77).

## 4 Lipid metabolism as a therapeutic target for MM

### 4.1 Current treatment modalities targeting lipid metabolism

Statins, by inhibiting HMG - CoA reductase in the mevalonate pathway, block the biosynthesis of cholesterol and isoprenoid intermediates (78–85). Preclinical studies, such as those on simvastatin, have demonstrated that it can induce mitochondrial dysfunction and endoplasmic reticulum stress in MM cells, subsequently activating the caspase - 3 - mediated apoptotic pathway. This interference with the MM cell membrane integrity and Ras/Rho protein is oprenylation disrupts normal cell functions, subjecting MM cells to metabolic stress and eventually leading to apoptosis (86). FASN, a key enzyme in fatty acid *de novo* synthesis highly expressed in MM cells, when inhibited, leads to reduced palmitate synthesis, disrupted lipid raft structure, and impaired pro - survival signaling like the AKT/mTOR pathway. Additionally, it induces ROS accumulation and an imbalance in the Bax/Bcl - 2 ratio, ultimately triggering mitochondria - dependent apoptosis and imposing significant metabolic stress on MM cells (87). The PI3K/Akt/mTOR pathway, which is well - known for its role in lipid metabolism, affects this process in multiple ways. For example, Akt can activate fatty acid synthase (FASN) and increase lipid uptake by cells. Conversely, mTOR complex 1 (mTORC1) can inhibit fatty acid β - oxidation, a catabolic process, by inhibiting AMP - activated protein kinase (AMPK) and its downstream targets, including PGC - 1α and SIRT1. Consequently, therapeutic strategies targeting this pathway have been extensively studied, leading to the development of several drugs. These drugs, commonly referred to as PI3K inhibitors, Akt inhibitors, or mTOR inhibitors depending on their position in the pathway, can inhibit one or more enzymes in the pathway, disrupting signaling and inhibiting the growth and survival of cancer cells. For instance, mTOR inhibitors like rapamycin and its derivatives, known as rapalogs, can block mTOR enzyme activity, thereby reducing protein synthesis and cell growth. These drugs have demonstrated clinical effects in certain cancers, such as kidney and breast cancer, and are currently under investigation for MM and other cancers. For example, silymarin has been found to induce apoptosis in MM cells through the PI3K/Akt/mTOR signaling pathway (88), and AE - 848, a novel small molecule compound, effectively induces apoptosis in human MM cells by regulating NF - κB and PI3K/Akt/mTOR signaling pathways (89).CPT1 inhibitors act by blocking fatty acid oxidation through inhibiting CPT1. This results in ATP depletion and AMPK hyperactivation in MM cells, inducing the transition from autophagy to apoptosis. By interfering with lipid metabolic homeostasis, these drugs cause MM cells to experience an energy crisis, oxidative stress, and membrane signaling disorders, all of which contribute to the induction of cell death (10).

By interfering with lipid metabolic homeostasis, these drugs cause MM cells to experience an energetic crisis, oxidative stress (ROS accumulation), and membrane signaling disorders. Eventually, they induce cell death via endogenous apoptotic pathways or exogenous death receptor pathways. In addition, metabolic stress enhances the sensitivity of MM cells to proteasome inhibitors, indicating the potential for co - administration (90).

### 4.2 Combination therapies targeting lipid metabolism

Combining drugs targeting lipid metabolism with other medications represents a highly promising treatment approach. For the combination of lipid - targeted drugs and proteasome inhibitors, examples like simvastatin (a statin) combined with bortezomib (a proteasome inhibitor) show that simvastatin inhibits HMG - CoA reductase (91), disrupts the lipid raft structure of MM cell membranes, and interferes with survival signaling pathways, while bortezomib induces endoplasmic reticulum stress and apoptosis, and the combination can enhance the sensitivity of endoplasmic reticulum stress induced by bortezomib and overcome drug resistance; In another study, the combination of lovastatin and MG-132 was found to be synergistically cytotoxic to MM.1S and RPMI8226 cells. The addition of lovastatin also increased the percentage of apoptotic cells compared to MG-132 alone. These results suggest that PI-induced lipid accumulation may confer acquired resistance to MM cells and, therefore, lovastatin may exert a synergistic anti-tumor proliferative effect with PI by reducing intracellular lipid levels (92).

In the treatment of multiple myeloma, significant progress has been made with monoclonal antibodies and immunotherapy. However, instances where lipid-targeted drugs are combined with these therapies remain relatively rare. In recent years, the application of monoclonal antibodies in multiple myeloma has become a research hotspot. Particularly, monoclonal antibodies targeting CD38 and SLAMF7, such as daratumumab (daratumumab) and ibrutinib (elotuzumab), have shown promising efficacy in patients with relapsed/refractory multiple myeloma (RRMM) (93). In addition, bispecific antibodies (BsAbs), as a novel immunotherapy approach, have shown potential in the treatment of multiple myeloma. BsAbs can bind to both the CD3 subunit of T-cell receptor complexes and antigens on tumor cells, thereby activating T cells and killing tumor cells (94). These antibodies have demonstrated high overall response rates (ORRs) in clinical trials, and efforts are actively underway to improve their efficacy and tolerability.

Despite this, research combining lipid-targeted drugs with monoclonal antibodies or immunotherapies is still in its infancy. Existing studies primarily focus on the combination of monoclonal antibodies and immunotherapies, such as CAR-T cell therapy and bispecific antibodies, which show promising prospects in the treatment of multiple myeloma (95). Future research may explore the synergistic effects of lipid-targeted drugs with these immunotherapies to enhance therapeutic outcomes and reduce side effects.

### 4.3 Emerging strategies for the treatment of multiple myeloma

Based on ongoing trials or novel compounds targeting lipid metabolism, as shown in **Table 1**, there are currently multiple drugs with diverse mechanisms of action and trial statuses being explored for the treatment of multiple myeloma (MM).

**Table 1:** Lipid metabolism-targeted therapies for multiple myeloma.

Drugs	Targets/Pathways	Mechanism	Current status of the trial
Opaganib (96)	SphK	Inhibiting SphK2 reduces the production of sphingosine - 1 - phosphate (S1P), induces apoptosis of multiple myeloma (MM) cells and enhances the sensitivity of MM cells to proteasome inhibitors.	Phase I clinical trial has initially demonstrated safety and potential efficacy.
TVB-2640 (97)	FASN	Inhibit FASN, block fatty acid synthesis, induce endoplasmic reticulum stress and apoptosis of MM cells, and overcome drug resistance.	Phase I/II trials are underway, evaluating the efficacy of combination with other drugs.
Statins (98)	3-Hydroxy-3-Methylglutaryl CoenzymeA Reductase	Inhibit the mevalonate pathway and reduce cholesterol synthesis, which may inhibit the proliferation of MM cells by regulating the Ras/MAPK pathway.	Retrospective clinical studies have shown that combination therapy prolongs survival; there are no dedicated Phase III trials yet.
Idelalisib (99)	PI3K	Inhibit the PI3K/Akt/mTOR pathway and block the survival and proliferation signals of MM cells.	Early trials were terminated due to limited efficacy and immune - related toxicity.
Elesclomol (100)	Iron metabolism/Lipid peroxidation	Induce the accumulation of reactive oxygen species (ROS), promote lipid peroxidation, and lead to ferroptosis of MM cells.	Pre - clinical studies have shown that the combination with bortezomib is effective; however, it has not yet entered clinical trials for multiple myeloma (MM).

Opaganib exerts its action by specifically targeting Sphingosine Kinase (SphK). Through the inhibition of SphK2, it effectively reduces the production of Sphingosine - 1 - Phosphate (S1P). This dual - effect not only induces apoptosis in multiple myeloma (MM) cells but also significantly enhances their sensitivity to proteasome inhibitors. The initial results of its Phase I clinical trial have provided evidence of both safety and potential efficacy (96).TVB - 2640 is centered on Fatty Acid Synthase (FASN). By inhibiting FASN, it blocks the process of fatty acid synthesis, thereby triggering endoplasmic reticulum stress and apoptosis in MM cells. Moreover, it has the capacity to overcome drug resistance (97). Currently, Phase I/II clinical trials are being carried out to comprehensively evaluate its combination with other drugs. Statins act on 3 - Hydroxy - 3 - Methylglutaryl Coenzyme A Reductase, thereby suppressing the mevalonate pathway and cholesterol synthesis. They potentially impede the proliferation of MM cells by regulating the Ras/MAPK pathway. Although retrospective clinical studies have demonstrated that combination therapy can extend the survival of MM patients (98) dedicated Phase III clinical trials are still lacking.Idelalisib targets Phosphoinositide 3 - Kinase (PI3K), inhibiting the PI3K/Akt/mTOR pathway to disrupt the survival and proliferation signals of MM cells (99). However, early - stage clinical trials were terminated due to limited efficacy and immune - related toxicity. Elesclomol impacts iron metabolism and lipid peroxidation. It induces the accumulation of Reactive Oxygen Species (ROS), promotes lipid peroxidation, and ultimately causes ferroptosis in MM cells (100). Pre - clinical studies have indicated that its combination with bortezomib shows effectiveness, yet it has not yet entered the stage of MM clinical trials.

These drugs represent a significant step forward in the development of novel therapies for MM. They hold great transformative potential as they target key aspects of MM cell biology, especially lipid metabolism. However, challenges remain, such as optimizing dosing regimens, minimizing side - effects, and determining the best combination strategies. Overcoming these challenges will be crucial to translate these promising findings into effective treatments for MM patients.

## 5 Potential clinical applications and future perspectives

### 5.1 Integrating new technologies for in-depth understanding

Lipidomics, a powerful systems - biology approach, enables researchers to comprehensively analyze lipid molecule types and their intricate metabolic pathways within cells, tissues, or entire organisms. In the field of multiple myeloma (MM), its significance cannot be overemphasized. MM cells are characterized by abnormal lipid metabolism, which is intricately linked to tumor cell proliferation, survival, and the thorny issue of drug resistance. Through meticulous lipidomic analysis, specific lipid metabolites and pathways closely related to MM, such as sphingolipids, glycerophospholipids, and cholesterol metabolism pathways, can be precisely identified (101). These identifications are not only crucial for understanding the disease mechanism but also serve as potential therapeutic targets, laying the foundation for the development of lipid - targeted therapies.

Artificial Intelligence (AI) technologies are making remarkable progress in the medical field, especially in the crucial area of predicting patient responses to specific treatments. By seamlessly integrating patients’ genomic, lipidomic, and clinical data, AI can construct highly sophisticated predictive models. For example, machine - learning algorithms can sift through vast amounts of lipidomics data to identify biomarkers associated with treatment responses. This newly - acquired ability to predict which patients are more likely to benefit from lipid - targeted therapies has significantly propelled the development of personalized medicine, bringing us closer to the goal of tailoring treatment plans for MM patients.

The combined power of lipidomics and AI provides a potent toolkit for biomarker discovery and patient stratification in MM patients. Lipidomics can accurately pinpoint specific lipid metabolites or pathways that are dysregulated in MM. AI - driven analysis then uses these findings to stratify patients into distinct subgroups. Some lipid metabolites can act as reliable indicators of high sensitivity to specific lipid - targeted therapies, while others may be closely associated with drug resistance (102, 103). This patient stratification is of great significance for optimizing treatment plans. Clinicians can formulate treatment plans based on the characteristics of individual patients, achieving more effective and efficient treatment.

### 5.2 Confronting the challenges

One of the most pressing challenges in lipid - targeted therapies is ensuring high treatment specificity. Minimizing off - target effects on normal tissues is of utmost importance. Since lipid metabolism is a fundamental process in all cells, developing therapies that can specifically target the abnormal lipid metabolism in MM cells without disrupting the normal functions of normal cells is a formidable task. Scientists are actively exploring various strategies, such as designing drugs with high - affinity binding to the specific lipid pathways or metabolites that are dysregulated in MM cells. Nanoparticle - based drug delivery systems are also under intensive investigation to enhance the specific delivery of lipid - targeted drugs to MM cells, thereby reducing the impact on normal tissues. Drug resistance is a major obstacle in the treatment of MM, especially in lipid - targeted therapies. MM cells often develop resistance over time, leading to disappointing treatment outcomes. Combination therapies present a promising solution. By combining lipid - targeted drugs with other therapeutic agents, such as proteasome inhibitors, monoclonal antibodies, or immunotherapies, we can simultaneously target multiple aspects of MM cell survival mechanisms, reducing the likelihood of cells developing drug resistance. In addition, adaptive therapies that target the metabolic plasticity of MM cells are also being studied. These therapies can adapt to the dynamic changes in the lipid metabolism of MM cells, ensuring the continuous effectiveness of treatment.

There is an urgent need for large - scale research to validate the efficacy of lipid - metabolism - based interventions. Well - designed clinical trials involving a large number of patients are crucial for accurately evaluating the true potential of lipid - targeted therapies. These trials should not only focus on treatment outcomes but also closely monitor the safety and long - term effects of these therapies. Large - scale research can provide valuable insights into the optimal dosage, treatment duration, and combination strategies of lipid - targeted therapies. Integrating lipid - targeted therapies into the framework of personalized medicine is of great significance. Using lipidomics and AI for biomarker discovery and patient stratification helps to formulate highly personalized treatment plans. By understanding the unique lipid metabolism characteristics of individual patients, clinicians can make more informed choices about the most suitable lipid - targeted therapies. This personalized approach can lead to better treatment outcomes, reduce side effects, and ultimately improve the quality of life of MM patients.

## 6 Conclusion

In conclusion, the current body of research on the role of lipid metabolism in multiple myeloma (MM) has firmly established its fundamental importance in the pathogenesis and progression of the disease. Imbalanced lipid metabolism in MM is a complex phenomenon, characterized by alterations in lipogenesis, disruptions in lipid uptake and trafficking, and perturbations within the tricarboxylic acid (TCA) cycle. These metabolic aberrations drive the reprogramming of energy metabolism, biomass production, and redox homeostasis within MM cells. Concurrently, the aberrant expression and activity of key lipid metabolism enzymes and transporters have been identified, underscoring the pivotal role of this imbalances in MM’s development and making lipid metabolism an enticing therapeutic target.

This imbalances not only enables MM cell survival, proliferation, and drug resistance but also exerts a profound influence on the tumor microenvironment (TME) and immune responses. For instance, lipid - induced oxidative stress in the TME can damage cellular components in MM cells and impact immune cell function, while lipid - rich tumor - associated macrophages (TAMs) promote immune evasion through multiple mechanisms such as secreting immunosuppressive factors and expressing immune checkpoint molecules.

Targeting lipid metabolism has emerged as a highly promising approach for MM treatment. Strategies like inhibitors of lipogenesis, lipid uptake, and the modulation of the TCA cycle, as well as those involving the inhibition of fatty acid synthesis, promotion of fatty acid oxidation, modulation of lipid signaling pathways, modification of the bone marrow (BM) microenvironment, and regulation of lipid - mediated communication within the TME, have shown great potential in preclinical studies and early - phase clinical trials. These approaches hold the potential to enhance patient outcomes by selectively eliminating MM cells, overcoming drug resistance, and minimizing adverse effects. Additionally, lipid - based biomarkers and metabolic imaging techniques, when combined with artificial intelligence, offer new tools for more accurate diagnosis, prognosis, and treatment response prediction, and can assist in patient stratification for personalized treatment. Meanwhile, in multiple myeloma (MM), angiogenesis and immunosuppression are two key features of the tumor microenvironment (TME). Therapeutic strategies targeting vascular endothelial growth factor (VEGF) and hepatocyte growth factor (HGF) have shown potential in disrupting these processes. VEGF is an important angiogenic factor that plays a crucial role in the progression of multiple myeloma. By inhibiting VEGF, the formation of tumor blood vessels can be reduced, thereby limiting tumor growth and spread (104). In addition, the HGF/MET pathway is also considered an important therapeutic target in multiple myeloma. HGF not only promotes the proliferation and survival of tumor cells but also increases angiogenesis and immunosuppression through interactions with the microenvironment. Abnormal activity of this pathway is associated with poor patient outcomes; therefore, targeting the HGF/MET pathway may help improve treatment efficacy (105). By targeting both VEGF and HGF simultaneously, the tumor immune microenvironment (TIME) can be more effectively reshaped to enhance the immune system’s response to tumors. Dual suppression promotes immune activation and complements immunotherapies such as anti-PD-1 or anti-BCMA (55).This strategy not only helps inhibit tumor angiogenesis but may also boost the effectiveness of immunotherapy by reducing the accumulation of suppressor cells (106). Therefore, combining anti-angiogenic and immunomodulatory treatments could provide a more comprehensive treatment approach for multiple myeloma patients. Integrating with lipid metabolism - related targets could offer a highly promising strategy for achieving long - term and effective disease control.

However, translating lipid metabolism research into clinical practice is fraught with challenges. The intricate nature of lipid metabolism pathways, the high degree of heterogeneity in MM, and the complex interplay between lipid metabolism and the TME pose significant obstacles. Ensuring the selectivity and specificity of treatments to target malignant plasma cells while sparing normal cells, managing drug resistance, and optimizing drug delivery systems are crucial steps for the successful implementation of lipid - metabolism - targeted therapies. Looking forward, future research should be dedicated to unraveling the complex mechanisms underlying lipid metabolism in MM and identifying novel therapeutic targets. Combination therapies that integrate lipid - metabolism - targeted agents with existing treatments, immunotherapeutic approaches, and personalized medicine strategies hold great promise for improving patient outcomes and surmounting drug resistance. The integration of lipid metabolism profiling into clinical decision - making processes will be essential for tailoring treatment strategies to individual patients.

In summary, the study of lipid metabolism in MM has provided invaluable insights into the disease’s biology and therapeutic potential. Targeting the dysregulated lipid metabolism pathways in MM cells and their interactions within the TME represents a highly promising avenue for future research and clinical interventions. Sustained efforts in this field will undoubtedly contribute to the development of innovative treatment strategies and, ultimately, improved outcomes for patients with MM.
